# Source and level of dietary iron influence semen quality by affecting inflammation, oxidative stress and iron utilization levels in boars

**DOI:** 10.1186/s40104-024-01032-5

**Published:** 2024-07-06

**Authors:** Yinghui Wu, Yamei Li, Yueyue Miao, Hongkui Wei, Hefeng Luo, Chunxiao Ren, Yawei Zhang, Juan Chen, Tanghong Wei, Jiyan Deng, Jian Peng

**Affiliations:** 1 Department of Animal Nutrition and Feed Science, College of Animal Science and Technology, Huazhong Agricultural University, Wuhan, 430070 China; 2 The Cooperative Innovation Center for Sustainable Pig Production, Wuhan, 400700 China; 3 Frontiers Science Center for Animal Breeding and Sustainable Production, Wuhan, 430070 China; 4 Dekon Food and Agriculture Group, Chengdu, 610000 China; 5 Xingjia Bio-Engineering Co. Ltd, Changsha, 410011 China

**Keywords:** Adult boars, Iron level, Iron source, Iron status, Semen quality

## Abstract

**Background:**

Boars fed a mixed form of inorganic and organic iron in excess of the NRC recommended levels still develop anemia, which suggested that the current level and form of iron supplementation in boar diets may be inappropriate. Therefore, 56 healthy Topeka E line boars aged 15–21 months were randomly divided into 5 groups: basal diet supplemented with 96 mg/kg ferrous sulfate (FeSO_4_) and 54 mg/kg glycine chelated iron (Gly-Fe, control); 80 mg/kg or 115 mg/kg Gly-Fe; 80 mg/kg or 115 mg/kg methionine hydroxyl analogue chelated iron (MHA-Fe, from Calimet-Fe) for 16 weeks. The effects of dietary iron supplementation with different sources and levels on semen quality in boars were investigated.

**Results:**

1) Serum Fe and hemoglobin concentrations were not affected by reduced dietary iron levels in the 80 mg/kg or 115 mg/kg Gly-Fe and MHA-Fe groups compared with the control group (*P >* 0.05). 2) Serum interleukin-6 (IL-6) and sperm malondialdehyde (MDA) levels in the 80 mg/kg or 115 mg/kg MHA-Fe groups were lower than those in the control group (*P* < 0.05), and higher serum superoxide dismutase levels and lower MDA levels in the 115 mg/kg MHA-Fe group (*P* < 0.05). 3) Boars in the 80 mg/kg or 115 mg/kg Gly-Fe and MHA-Fe groups had lower serum hepcidin (*P* < 0.01), ferritin (*P* < 0.05), and transferrin receptor (*P* < 0.01) concentrations, and boars in the 115 mg/kg MHA-Fe group had higher seminal plasma Fe concentrations compared with the control group. 4) Boars in the 80 mg/kg and 115 mg/kg MHA-Fe groups had lower abnormal sperm rate and in situ oscillating sperm ratio compared to the control group at weeks 12 and/or 16 of the trial. However, the effect of Gly-Fe on improving semen quality in boars was not evident. 5) Serum IL-6 level was positively correlated with hepcidin concentration (*P* < 0.05), which in turn was significantly positively correlated with abnormal sperm rate (*P* < 0.05). Furthermore, significant correlations were also found between indicators of iron status and oxidative stress and semen quality parameters.

**Conclusions:**

Dietary supplementation with 80 mg/kg or 115 mg/kg MHA-Fe did not induce iron deficiency, but rather reduced serum inflammatory levels and hepcidin concentration, alleviated oxidative stress, increased body iron utilization, and improved semen quality in adult boars.

## Introduction

The popularization of artificial insemination technology has made the importance of boar semen quality more and more prominent. However, the proportion of boars culled annually due to poor semen quality is as high as 23.7%–45.1% [1–3], which seriously reduces the return on investment of boars [1]. Iron (Fe) is one of the most abundant essential trace elements in the animal body. It plays a crucial role in spermatogenesis and semen quality by participating in physiological processes such as oxygen transportation, cellular metabolism, and DNA synthesis in the body [4]. In the previous study, we observed that the serum Fe level of Duroc boars (1.11 mg/L vs. 1.24 mg/L) and Yorkshire boars (1.11 mg/L vs. 1.34 mg/L) with poor semen quality was lower than that of boars with excellent semen quality, and the serum Fe level was positively correlated with the sperm motility and/or negatively correlated with the abnormal sperm rate [5, 6]. This suggests that an adequate level of Fe in the body is a guarantee of excellent semen quality in boars. The United States National Research Council (NRC) [7] and the Chinese Nutritional Requirements for Swine [8] recommend a dietary iron level of 80 mg/kg for boars. However, in practice, the dietary iron supplementation for boars typically ranges from 1.29 to 2.96 times the NRC recommendation, usually in the form of ferrous sulfate (FeSO_4_) and/or glycine-chelated iron (Gly-Fe), among others [9, 10]. Notably, our investigation of the body iron status of boars at artificial insemination stations showed that when 160 mg/kg mixed iron sources (80 mg/kg FeSO_4_ and 80 mg/kg Gly-Fe) were added to the basal diet (containing 111.63 mg/kg Fe) as a source of dietary iron for boars, 4.40% of the boars still were anaemic (hemoglobin level less than 90 g/L) and 46.88% of the boars were clinically anaemic (hemoglobin level 90–110 g/L). More importantly, anemic and clinically anaemic boars had significantly lower serum iron levels and impaired semen quality (unpublished data). Therefore, it is worth investigating whether the source and level of dietary iron should be adjusted to increase total body iron levels or iron levels used for spermatogenic functions to improve boar semen quality.

Since DMT1, a divalent metal ion transporter responsible for intestinal Fe absorption, can also be used to transport divalent metal ions such as Cu and Mn [11], pig producers usually choose to add high levels of Fe to the diet to ensure adequate Fe uptake, taking into account that Fe and Cu and Mn are antagonistic in the body’s absorption and that the cost of adding minerals to the diet is low compared to the total cost of the feed [9]. However, DMT1 has been shown to specifically transport Fe more efficiently than other elements (Fe^2+^ > Co^2+^, Mn^2+^ >> Zn^2+^) [11]. In addition, Fe ions can also be transported by Ctr1, a Cu ion transporter channel [12, 13]. This suggests that the organism may not require excessive Fe intake. More importantly, it has been known that FeSO_4_ has lower absorption rate and is structurally unstable, which tends to produce a large amount of free Fe^2+^ [14]. An increased level of Fe^2+^ promotes the production of reactive oxygen species (ROS) and induces elevated levels of inflammatory cytokines, such as interleukin-6 (IL-6) and tumor necrosis factor-α (TNF-α) [15]. These cytokines stimulate the secretion of hepcidin and inhibit iron absorption [16], which may result in boars having insufficient iron levels despite the inclusion of Fe beyond their nutritional requirements. Based on this, the selection of structurally stable amino acid chelated iron as a dietary source of iron may be an important measure to reduce the inflammatory response and hepcidin levels and improve semen quality in boars. Unfortunately, the effects of dietary iron sources and levels on reproductive performance in boars are poorly understood. A review of the PIC Nutrition and Feeding Guideline [17] and other recommended standards for the addition of iron to boar diets showed that the recommended amount of iron is between 80 and 100 mg/kg, which suggested that iron from appropriate sources may meet the boar’s needs at a lower level than 160 mg/kg used in production.

Therefore, in this study, the minimum dietary requirement of 80 mg/kg of iron in boar diets and the median of 115 mg/kg of iron supplementation in related swine diet studies were selected as the experimental iron levels, and the effects of Gly-Fe and methionine hydroxyl analogue chelate iron (MHA-Fe) on the body iron status and semen quality were investigated. The objectives of this study were to determine the appropriate source and level of iron to improve semen quality in adult boars, to provide a reference for iron supplementation in actual production.

## Methods

### Animals and experimental design

All animal research procedures were conducted in accordance with animal research guidelines issued by the Institutional Animal Care and Use Committee of Huazhong Agricultural University. A total of 56 healthy adult Topeka E line boars aged 15–21 months were selected and serum samples were collected prior to the start of the test and rapidly returned to the laboratory for determination of serum Fe content. In addition, semen quality information was collected from the boars in the 2 months prior to the test, and the average semen quality and serum Fe content of each boar was counted, and the boars were divided into five test groups with no difference in serum Fe content and semen quality according to the principle of consistency between serum Fe content and semen quality. The treatments consisted of (1) control (54 mg/kg Gly-Fe and 96 mg/kg FeSO_4_), (2) 80 mg/kg Gly-Fe, (3) 115 mg/kg Gly-Fe, (4) 80 mg/kg MHA-Fe, (5) 115 mg/kg MHA-Fe. The Fe content of Gly-Fe (Gly-Fe, Xingjia Biological Engineering Co., Ltd., Changsha, China) and MHA-Fe (Calimet-Fe, Xingjia Biological Engineering Co., Ltd., Changsha, China) was 18% and 12%, respectively, and dietary Fe supplementation was measured by Fe content and not by total ferrous sulphate or amino acid chelated iron content. Each group consisted of 10–12 boars, with each boar considered as a replicate, and the test period lasted for 16 weeks. Blood and semen samples were collected from boars at weeks 0, 4, 8, 12, and 16 of the test period. Blood samples were used for analyzing serum Fe and hemoglobin levels at all time points, while serum iron metabolism indices, inflammatory factor levels, and oxidative stress levels were analyzed only at week 16. Semen samples were analyzed for routine semen quality at all time points, and additional analysis of sperm motility and morphological parameters was conducted at weeks 8, 12, and 16. Boar semen was collected at a frequency of 3 times every 2 weeks. Boars were housed individually on slatted flooring (2.4 m × 0.8 m) and fed 2.5 kg of feed per day, divided into 2 feeding periods (07:00 and 14:00), with free access to water. The boar house was equipped with a positive pressure ventilation system, an automatic feeding system and a thermostatic control system (Automated Production Systems, AGCO Corporation, Illinois, USA). The room temperature during the experiment was 18.8–26.9 °C. All other feeding management and immunization protocols of boars were according to the same regulations of the farm. The boar test ration composition and nutrient levels are shown in Table 1, and the content of Fe in base diet was 116.85 mg/kg.

**Table 1:** Composition and nutrient levels of basal diets (air-dry basis)

Items	Content
Ingredients, %	
Wheat	40.00
Corn	17.00
Flour	15.00
Soybean meal	5.10
Bran	15.90
Limestone	1.08
CaHPO_4_	1.78
NaCl	0.50
Other components	1.64
Vitamin and mineral premix^a^	2.00
Total	100
Nutrient levels^b^	
DE, Mcal/kg	3.05
Crude protein, %	14.00
Calcium, %	0.90
Total phosphorus, %	0.87
Available phosphorus, %	0.45
Lysine, %	0.83
Methionine, %	0.34
Threonine, %	0.65
Tryptophan, %	0.17

^a^The premix provided the following per kg of diets: 5 mg Cu, 50 mg Zn, 20 mg Mn, 0.14 mg I, 0.30 mg Se, 15,000 IU vitamin A, 2,400 IU vitamin D_3_, 50 µg 25(OH)D, 0.48 mg menadione, 2 mg thiamin, 7.2 mg riboflavin, 3.6 mg pyridoxine, 25 µg vitamin B_12_, 0.48 mg biotin, 25 mg pantothenic acid, 4 mg folic acid, 400 mg niacin

^b^*DE* Digestible energy; Digestible energy and available phosphorus were calculated values, others were measured values

### Serum, seminal plasma and sperm Fe concentration

Venous blood samples were collected from either the left or right hind leg of each boar into 10-mL tubes without anticoagulant. The samples were then centrifuged at 1,800 × *g* for 10 min at 4 °C to obtain serum, which was subsequently divided and stored at –20 °C. At the time of sampling, the test boars were caught up to the dummy sow station and the boar semen was collected using the freehand semen collection method and placed in the semen collection bag. The fresh semen was then transported to the semen quality testing laboratory. In the laboratory, 5 mL of semen was aspirated from each boar into a 10-mL centrifuge tube and the semen was centrifuged at 800 × *g* for 10 min to separate the seminal plasma, which was stored at –20 °C. Subsequently, the bottom sperm precipitate was washed three times with phosphate buffer saline, the sperm samples were divided into 1 mL per tube and stored at -20°C. The samples were transferred to –80 °C for storage as soon as possible. For the determination of Fe concentration in serum, seminal plasma and sperm, the serum, seminal plasma and sperm samples were pre-treated by water bath digestion, and then detected by inductively coupled plasma mass spectrometer (Agilent 7900, Agilent Technologies, Inc., California, USA). Among them, the results of Fe elemental determination in sperm were corrected by the sperm density of the current semen, and the Fe elemental content in the boar’s sperm sample was expressed in terms of the content per 10^6^ cell samples.

### Hemoglobin concentration

Blood was collected from either the left or right hind leg of the boar while it remained calm, without feeding or insemination. Hemoglobin levels were measured using a portable hemoglobin analyser (HemoCue^®^ Hb 201^+^, Danaher company, Washington, USA), and the average of three hemoglobin readings for each boar was calculated as its hemoglobin level.

### Serum iron metabolism indices

Total iron binding capacity was determined using the corresponding assay kits (A040-1-1, Nanjing Jiancheng Bioengineering Institute, Nanjing, China); serum hepcidin (CQ100993), ferritin (CQ101626) and transferrin receptor (ml025371) were determined using ELISA assay kits (Shanghai Qifa Experimental Reagent Co., Ltd., Shanghai, China).

### Semen quality parameters

#### Conventional semen quality parameters

Ejaculate volume: semen weight was measured using an electronic balance and converted to semen volume (approximately 1 g = 1 mL).

Sperm density: measured by sperm densitometer (SDM1, Minitube International, Diefenbach, Germany).

Sperm motility: fresh semen was diluted 1:9 with semen diluent and 10 µL of the mixed diluted semen sample was applied to a pre-warmed slide at 37 °C. The prepared slide was then placed under a light microscope with a magnification set to 200×, and the sperm within the field of view were observed for spontaneous motility with the naked eye. Viable sperm typically exhibit rapid, linear movement, while inactive sperm remain stationary. The total number of sperm in the field of view and the number of viable sperm were counted to determine the sperm motility in that particular field. To ensure statistical reliability, five randomly selected fields of view were observed for each boar’s semen sample, ensuring a minimum count of 200 sperm. The mean value of sperm motility across these fields of view was then calculated and recorded as the sperm motility data for that boar.

Abnormal sperm rate: a slide containing 10 µL of diluted semen sample was placed under a 400× light microscope, and the sperm within the field of view were observed for normal morphology with the naked eye. The total number of sperm in the field of view and the number of sperm with abnormal morphology were counted, and the abnormal sperm rate was calculated accordingly. Five fields of view were randomly selected for observation of each boar’s semen, and information on at least 200 sperm was counted to calculate the mean value of the abnormal sperm rate.

$$\mathrm{Total}\;\mathrm{sperm}\;{\text{count:}}\;\mathrm{ejaculate}\;\mathrm{volume}\;\mathrm{of}\;\mathrm{the}\;\mathrm{current}\;\mathrm{ejaculation}\;\times\;\mathrm{sperm}\;\mathrm{density}.$$

$$\mathrm{Effective}\,\mathrm{sperm}\,{\text{count:}}\ \mathrm{total}\,\mathrm{sperm}\,\mathrm{count}\,\mathrm{of}\,\mathrm{the}\,\mathrm{current}\,\mathrm{ejaculation}\,\times\,\mathrm{sperm}\,\mathrm{motility}\,\times\,(1\ -\ \mathrm{abnormal}\,\mathrm{sperm}\,\mathrm{rate}).$$

#### Sperm motility parameters

A total of 10 µL of the diluted semen sample was automatically detected by a computer-assisted sperm testing system (CASA, AndroVision^®^, Minitube International, Diefenbach, Germany) to determine the spermatozoa ratio of forward-moving sperm, fast-moving sperm, slow-moving sperm, rotating sperm and in situ swinging sperm.

#### Sperm morphology parameters

The CASA system was used to detect abnormal sperm morphology, including the three main types of folded tails, distal protoplasmic droplets and proximal protoplasmic droplets.

### Inflammatory factor indicator assay

Serum levels of IL-6 (KMLJ941958p), interleukin-1β (IL-1β, KMLJ941941p), TNF-α (KMLJ942147p) and interleukin-10 (IL-10, KMLJ941944p) were determined by using kits (Nanjing Camillo Bioengineering Co., Ltd., Nanjing, China), and all test operations were performed strictly according to the kits.

### Detection of oxidative stress indicators

Superoxide dismutase (SOD), catalase (CAT), total antioxidant capacity (T-AOC), and malondialdehyde (MDA) levels in serum, seminal plasma, and sperm samples were determined using SOD (A001-3-2), CAT (A007-1-1), T-AOC (A015-2-1), and MDA (A003-1) kits (Nanjing Jiancheng Bioengineering Research Institute, Nangjing, China). All experimental procedures were strictly performed according to the requirements of the respective kits.

### Statistical analysis

Parametric statistical analyses were first performed using the Shapiro-Wilk test for the normal distribution characteristics of the data, for normally distributed data, the results are presented as mean ± standard deviation (standard error) in the presentation of the results, whereas the non-normally distributed data are presented as median [25^th^ quartile, 75^th^ quartile]. Between-group comparisons of trial data were conducted using the ANOVA process for normally distributed data and the Kruskal-Wallis process for non-normally distributed data. Correlation analysis between indicators was carried out statistically using the Pearson method for normally distributed data and the Spearman rank method for non-normally distributed data. Statistical analysis of all experimental data was performed using SAS 9.2 software. *P* < 0.05 for intergroup comparison indicated significant differences between groups, and *P* < 0.01 was considered highly significant; *P* < 0.05 for correlation coefficient of indicators in correlation analysis indicated significant correlation between two indicators, and *P* < 0.01 was considered highly significant correlation. Graphs in the results were plotted using Origin 8.0.

## Results

### No reduction in serum iron and hemoglobin levels in boars fed 80 mg/kg or 115 mg/kg Gly-Fe and MHA-Fe

**Fig. 1:**
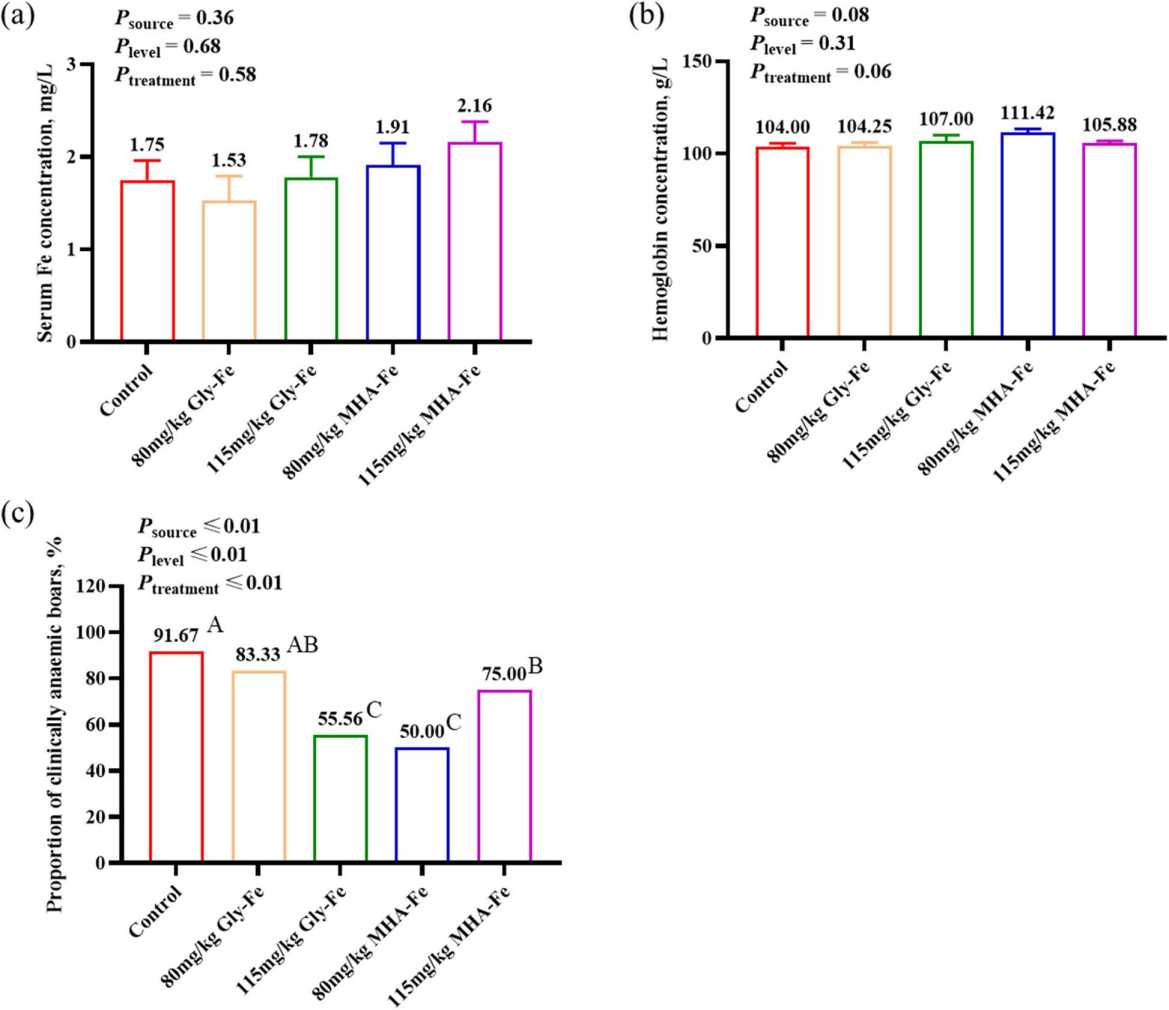
Effects of different iron sources and iron levels on iron levels in boars. **a** Serum Fe concentration; **b** Hemoglobin concentration. Data are presented as mean ± standard error. **c** Proportion of clinically anaemic boars. Hemoglobin levels below 110 g/L were considered to be clinically anaemic boars. ^A–C^Different letters indicate the highly significant difference between groups (*P* < 0.01)

Serum Fe concentration, which corresponds to the level of iron in the bloodstream, and hemoglobin concentration, which responds to iron deficiency anemia, were used to determine the effects of different sources and levels of iron supplementation on iron levels in boars. The results showed that supplementation with 80 mg/kg or 115 mg/kg Gly-Fe and 80 mg/kg or 115 mg/kg MHA-Fe, respectively, reduced the amount of iron in the diet but did not affect the serum iron concentration and hemoglobin concentration of the boars compared with the control group (*P* > 0.05; Fig. 1a and b). Hemoglobin levels below 90 g/L were considered anemic boars and below 110 g/L were considered clinically anemic boars. As all test pigs had hemoglobin levels above 90 g/L. Therefore, the proportion of clinically anemic boars in each treatment group was counted. It’s noteworthy that the percentage of clinical anemia was significantly lower among boars in the 115 mg/kg Gly-Fe group and those in the 80 mg/kg and 115 mg/kg MHA-Fe groups compared to the control group. The lowest percentage of clinical anemia was observed in boars from the 115 mg/kg Gly-Fe and 80 mg/kg MHA-Fe groups. This indicates that feeding low levels of Gly-Fe or MHA-Fe not only does not decrease the serum Fe and hemoglobin levels of boars but also reduces the proportion of clinically anemic boars.

### Lower levels of pro-inflammatory cytokine in boars fed 80 mg/kg or 115 mg/kg MHA-Fe

**Fig. 2:**
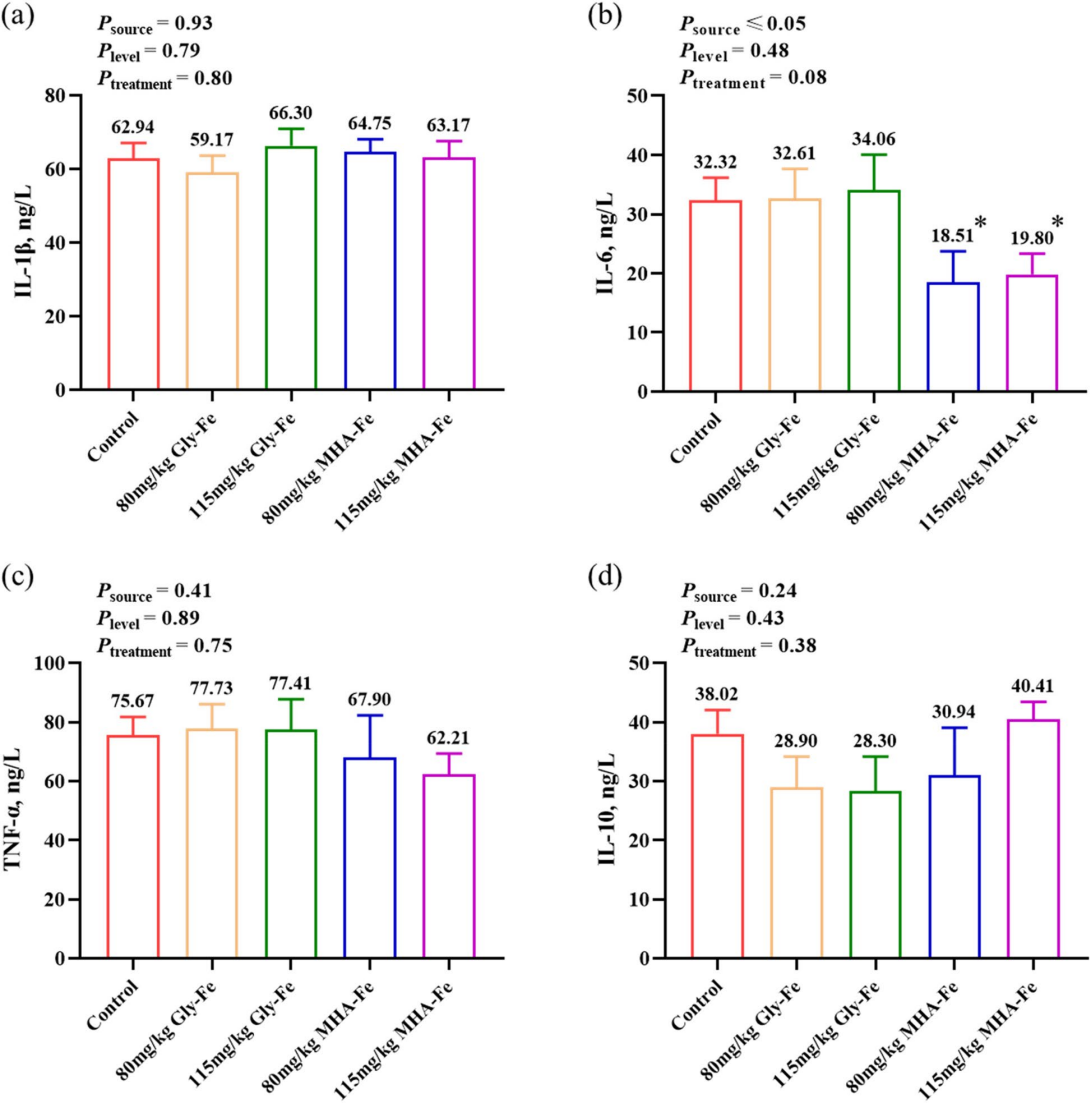
Serum inflammatory factor levels in boars. **a** IL-1β level; **b** IL-6 level; **c** TNF-α level; **d** IL-10 level. Data are presented as mean ± standard error. * indicates a significant difference compared with the control group (*P* < 0.05)

Serum levels of inflammatory factors are shown in Fig. 2a–d. At week 16 of the experiment, multiple group comparisons showed a trend towards a difference in serum levels of the pro-inflammatory cytokine IL-6 (*P* = 0.08). Serum IL-6 levels were significantly lower in boars in the 80 mg/kg or 115 mg/kg MHA-Fe groups compared with the control group (*P* < 0.05). The results of the analyses between groups of different iron sources also showed that serum levels of IL-6 were lower in boars in the MHA-Fe group than in the Gly-Fe group (*P* < 0.05) and the control group (*P* = 0.08). However, the dietary treatments had no significant effect on the levels of pro-inflammatory factors IL-1β, TNF-α and anti-inflammatory factor IL-10 (*P* > 0.05).

### Decreased oxidative stress levels in boars fed Gly-Fe or MHA-Fe

As shown in Table 2, the serum SOD content of boars in the 115 mg/kg MHA-Fe group was significantly higher than that of the control group (*P* < 0.05), and the serum MDA content of boars in the 115 mg/kg MHA-Fe group was the lowest among the five groups (*P* < 0.05). In addition, the MDA content in the sperm of boars in the 80 mg/kg Gly-Fe group, the 80 mg/kg and the 115 mg/kg MHA-Fe groups were also significantly lower (*P* < 0.01) than that of the control group. Unexpectedly, T-AOC levels in boar seminal plasma were lower in the 115 mg/kg Gly-Fe and 80 mg/kg MHA-Fe groups than in the control group (*P* < 0.01).

**Table 2:** Effects of different iron sources and levels on oxidative stress levels of boars^a^

Item	Control	Gly-Fe		MHA-Fe		*P*_source_^b^	*P*_level_^c^	*P*_treatment_^d^
		80 mg/kg	115 mg/kg	80 mg/kg	115 mg/kg			
Serum								
CAT, U/mL	3.94 ± 1.88	3.67 ± 1.19	3.75 ± 0.85	3.72 ± 1.04	5.45 ± 2.94	0.30	0.25	0.11
SOD, U/mL	50.82[42.84, 53.19]^B^	52.45[47.36, 56.74]^AB^	51.16[43.50, 59.37]^AB^	53.34[48.19, 59.31]^AB^	60.50[55.77, 63.23]^A^	< 0.05	0.07	< 0.05
T-AOC, mmol/L	0.87[0.83, 0.95]	0.86[0.82, 0.95]	0.80[0.75, 0.87]	0.80[0.76, 0.84]	0.89[0.87, 0.96]	0.25	0.25	0.28
MDA, nmol/mL	3.02[2.30, 3.81]^AB^	3.79[3.44, 4.16]^A^	3.17[2.71, 4.41]^AB^	2.77[1.68, 3.93]^AB^	2.32[1.95, 3.06]^B^	< 0.05	0.55	< 0.05
Seminal plasma								
CAT, U/mL	1.43 ± 0.95	1.25 ± 0.77	1.05 ± 0.48	1.55 ± 0.79	2.15 ± 1.14	< 0.05	0.77	0.12
SOD, U/mL	48.84[43.31, 61.90]	54.46[46.95, 62.15]	48.32[39.56, 59.00]	45.61[42.49, 61.33]	49.47[43.07, 56.17]	0.64	0.79	0.82
T-AOC, mmol/L	1.00[0.86, 1.10]^A^	0.83[0.69, 0.96]^AB^	0.54[0.36, 0.79]^B^	0.59[0.38, 0.79]^B^	1.20[0.81, 1.65]^A^	< 0.05	< 0.05	< 0.01
MDA, nmol/mL	1.14[1.06, 2.22]	1.46[0.93, 1.82]	1.22[1.09, 2.31]	2.04[1.62, 2.44]	1.38[1.35, 1.52]	0.55	0.78	0.47
Sperm								
CAT, U/mg	3.43 ± 2.31	2.97 ± 1.58	3.28 ± 1.67	3.01 ± 2.24	4.43 ± 1.86	0.63	0.35	0.60
SOD, U/mg	20.78[17.56, 25.23]	19.86[15.42, 24.08]	18.49[15.96, 30.11]	18.50[12.23, 28.42]	21.68[20.56, 30.35]	0.81	0.71	0.71
MDA, nmol/mg	2.32[1.55, 4.40]^A^	0.74[0.36, 1.11]^B^	1.34[0.00, 2.76]^AB^	0.67[0.00, 1.82]^B^	0.77[0.00, 1.52]^B^	< 0.05	< 0.05	< 0.05

^a^*CAT *Catalase, *SOD *Superoxide dismutase, *T-AOC *Total antioxidant capacity, *MDA *Malondialdehyde. serum, seminal plasma and sperm CAT concentrations were presented as mean ± standard deviation, whereas other data were presented as median [25^th^ quartile, 75^th^ quartile]

^b^Comparison between the control group, Gly-Fe groups and MHA-Fe groups

^c^Comparison between the control group, 80 mg/kg groups and 115 mg/kg groups

^d^Comparison between the control, 80 mg/kg Gly-Fe, 115 mg/kg Gly-Fe, 80 mg/kg MHA-Fe and 115 mg/kg MHA-Fe groups

^A,B^Different letters in the same row indicate significant differences in the indicators between the treatment groups (*P* < 0.05)

### Reduced body iron storage and increased iron utilization in boars fed Gly-Fe or MHA-Fe

**Fig. 3:**
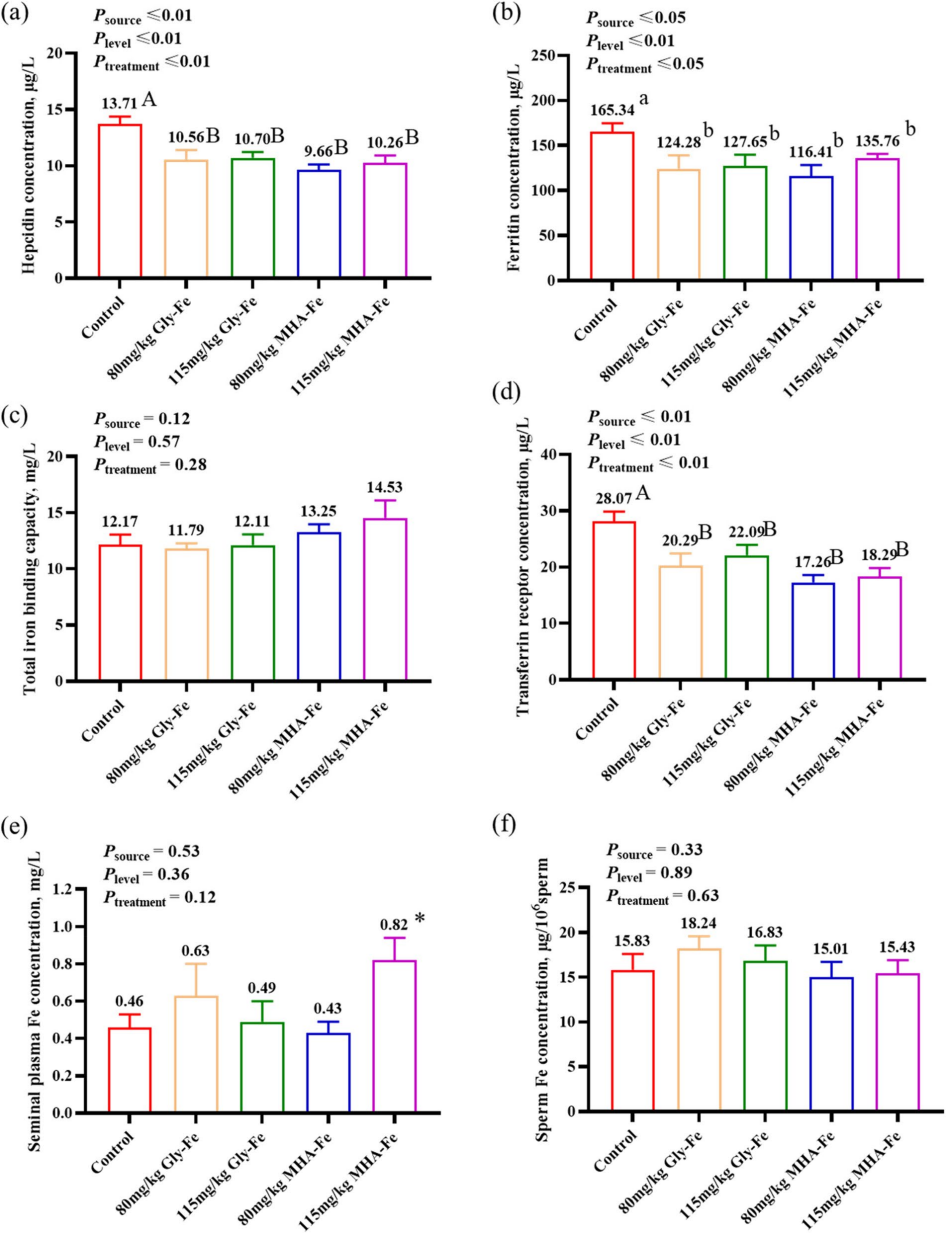
Effects of different iron sources and iron levels on iron status in boars. **a** Serum hepcidin concentration; **b** Ferritin concentration; **c** Total iron binding capacity level; **d** Transferrin receptor concentration; **e** Seminal plasma Fe concentration; **f** Sperm Fe concentration. Data are presented as mean ± standard error. ^a,b^Different letters indicate the significant difference between groups (*P* < 0.05); ^A,B^Different letters indicate the highly significant difference between groups (*P* < 0.01); * indicates a significant difference compared with the control group (*P* < 0.05)

The results of hepcidin, an indicator of body iron homeostasis regulated by inhibition of intestinal iron absorption, showed that the serum hepcidin concentration of boars in the 80 mg/kg or the 115 mg/kg Gly-Fe and MHA-Fe groups was significantly lower than that of boars in the control group (*P* < 0.01; Fig. 3a). Analysis of the indicator of iron storage showed that ferritin concentration was significantly lower in boars of the 80 mg/kg or the 115 mg/kg Gly-Fe and MHA-Fe groups compared with the control group (*P* < 0.05; Fig. 3b). The results of total iron binding capacity and transferrin receptor concentration, indicators of iron utilization in serum, showed that the transferrin receptor concentration in serum of boars in the 80 mg/kg or the 115 mg/kg Gly-Fe and MHA-Fe groups was lower than that of boars in the control group (*P* < 0.01). In addition, the differences in total iron binding capacity of boars between the different treatment groups were not significant (*P* > 0.05; Fig. 3c and d). It is also worth noting that there were no significant differences in serum hepcidin, ferritin and transferrin receptor levels in boars between the four groups: 80 mg/kg Gly-Fe, 115 mg/kg Gly-Fe, 80 mg/kg MHA-Fe and 115 mg/kg MHA-Fe. These results indicate that Gly-Fe or MHA-Fe fed boars had lower body iron stores, higher iron utilization and less inhibition of intestinal iron absorption by hepcidin.

Detection of Fe in seminal plasma and sperm revealed significantly higher concentrations of Fe in the seminal plasma of boars in the 115 mg/kg MHA-Fe group compared with the control group (*P* < 0.05; Fig. 3e). However, there was no significant difference in sperm Fe concentration between treatment groups (*P* > 0.05; Fig. 3f).

### Improved semen quality in boars fed 80 mg/kg or 115 mg/kg MHA-Fe

The effects of different treatments on conventional semen quality parameters of boars are presented in Table 3. The results showed that the abnormal sperm rate and functional sperm count were significantly affected by dietary iron source and level. Abnormal sperm rate of boars in the 80 mg/kg or 115 mg/kg MHA-Fe groups was significantly lower than that of boars in the control group at weeks 12 and 16 of the trial (*P* < 0.01). Functional sperm count was significantly increased in boars in the 115 mg/kg MHA-Fe group at week 16 of the trial compared to boars in the control group (*P* < 0.05). However, the difference in abnormal sperm rate and functional sperm count between boars in the Gly-Fe group and boars in the MHA-Fe and control groups were not significant. In addition, the effects of dietary treatments on semen volume, sperm concentration, sperm motility and total sperm count were not significant (*P* > 0.05).

**Table 3:** Effects of different iron sources and levels on conventional semen quality parameters of boars^a^

Item	Control	Gly-Fe		MHA-Fe		*P*_source_^b^	*P*_level_^c^	*P*_treatment_^d^
		80 mg/kg	115 mg/kg	80 mg/kg	115 mg/kg			
Ejaculate volume, mL								
Week 0	278.83 ± 63.13	283.08 ± 46.57	313.40 ± 85.89	275.08 ± 80.70	307.90 ± 46.63	0.75	0.23	0.56
Week 4	311.42 ± 97.89	322.33 ± 89.79	292.70 ± 124.11	313.67 ± 93.36	289.00 ± 66.72	0.96	0.63	0.91
Week 8	308.08 ± 72.30	291.08 ± 76.81	382.67 ± 74.79	347.33 ± 82.91	308.50 ± 63.07	0.70	0.42	0.06
Week 12	274.00 ± 64.34	291.92 ± 67.92	300.50 ± 107.75	304.45 ± 88.47	253.33 ± 45.14	0.72	0.56	0.60
Week 16	270.00 ± 51.86	277.67 ± 75.45	317.10 ± 134.52	267.55 ± 73.50	280.40 ± 79.22	0.61	0.53	0.69
Sperm concentration, ×10^8^/mL								
Week 0	3.14 ± 1.09	2.74 ± 0.87	2.98 ± 0.64	2.98 ± 1.01	2.90 ± 0.55	0.64	0.66	0.86
Week 4	3.33 ± 1.04	2.96 ± 0.65	3.39 ± 0.96	2.83 ± 0.80	3.31 ± 0.55	0.66	0.14	0.39
Week 8	3.30 ± 0.92	3.21 ± 0.99	2.67 ± 0.63	2.84 ± 0.82	3.13 ± 0.62	0.47	0.42	0.35
Week 12	3.50 ± 1.12	3.31 ± 0.83	3.08 ± 1.09	3.39 ± 1.60	3.97 ± 1.20	0.46	0.85	0.56
Week 16	3.39 ± 1.18	3.56 ± 0.91	3.31 ± 1.13	3.47 ± 1.25	3.80 ± 0.60	0.78	0.92	0.86
Sperm motility, %								
Week 0	82.00[81.00,83.00]	83.50[82.00,84.00]	83.00[80.00,84.25]	84.50[83.25,85.00]	81.00[80.00,84.00]	0.32	0.13	0.07
Week 4	88.00[81.25,90.00]	86.50[81.00,90.00]	83.50[81.50,88.00]	85.00[84.00,87.75]	87.00[84.50,90.00]	0.65	0.70	0.61
Week 8	86.00[81.00,90.75]	88.00[84.25,92.00]	83.50[81.50,84.75]	85.50[84.25,87.75]	83.50[80.75,87.25]	0.89	0.07	0.18
Week 12	86.00[80.75,90.00]	88.50[85.00,93.75]	85.00[83.50,88.00]	88.00[86.00,91.00]	89.50[85.00,91.25]	0.12	0.08	0.08
Week 16	90.00[81.00,90.00]	86.50[85.00,90.75]	88.50[86.75,90.75]	89.00[85.00,94.00]	90.50[86.25,93.25]	0.33	0.53	0.59
Abnormal sperm rate, %								
Week 0	18.68[14.00,21.16]	12.61[11.93,16.49]	18.82[15.80,22.17]	14.27[12.90,19.62]	16.18[11.73,19.05]	0.41	0.13	0.17
Week 4	14.86[12.14,21.58]	12.54[10.33,17.06]	14.91[11.81,25.68]	13.68[12.55,18.99]	16.48[12.17,21.43]	0.71	0.46	0.72
Week 8	15.64[12.42,18.56]	13.25[10.88,16.39]	14.77[13.03,19.55]	12.22[10.26,16.37]	12.43[8.82,15.36]	0.05	0.17	0.15
Week 12	21.79[17.16,37.01]^A^	14.72[12.23,22.26]^AB^	14.36[11.11,17.90]^AB^	13.81[11.74,17.35]^B^	11.20[10.13,16.36]^B^	< 0.01	< 0.01	< 0.01
Week 16	23.96[16.23,35.86]^A^	16.31[11.61,24.03]^AB^	14.61[12.89,17.91]^AB^	14.61[10.55,15.92]^B^	11.56[10.02,15.44]^B^	< 0.01	< 0.01	< 0.01
Total sperm count, ×10^9^/ejaculate								
Week 0	84.35 ± 23.83	76.55 ± 22.83	99.99 ± 29.92	78.84 ± 25.80	89.12 ± 21.37	0.92	0.10	0.24
Week 4	98.52 ± 30.85	92.85 ± 22.77	94.83 ± 41.83	85.94 ± 26.8	95.12 ± 24.42	0.85	0.80	0.97
Week8	107.65 ± 23.27	90.26 ± 27.88	99.63 ± 29.78	94.21 ± 19.04	95.10 ± 19.46	0.27	0.21	0.50
Week 12	101.80 ± 16.61	94.10 ± 21.47	81.46 ± 35.36	88.42 ± 50.91	104.10 ± 22.36	0.55	0.79	0.79
Week 16	96.01 ± 28.89	109.66 ± 37.82	93.30 ± 21.77	87.41 ± 19.58	119.96 ± 22.29	0.79	0.55	0.06
Functional sperm count, ×10^9^/ejaculate								
Week 0	55.85 ± 16.67	54.43 ± 18.51	70.17 ± 23.53	54.62 ± 14.93	60.61 ± 16.86	0.63	0.12	0.24
Week 4	70.61 ± 24.75	68.44 ± 18.07	65.35 ± 28.32	61.80 ± 16.33	67.00 ± 15.98	0.81	0.82	0.96
Week 8	69.57 ± 20.84	69.83 ± 23.34	60.41 ± 30.34	71.58 ± 16.37	69.42 ± 13.02	0.95	0.92	0.99
Week 12	61.38 ± 21.05	62.13 ± 16.43	70.78 ± 16.00	74.22 ± 36.39	76.02 ± 20.52	0.23	0.38	0.46
Week 16	61.19 ± 21.14^B^	77.57 ± 22.11^AB^	70.49 ± 17.90^AB^	67.31 ± 15.69^AB^	93.60 ± 14.54^A^	< 0.05	< 0.05	< 0.01

^a^Ejaculate volume, sperm concentration, total sperm count and functional sperm count were presented as mean ± standard deviation, whereas sperm motility and abnormal sperm rate were presented as median [25^th^ quartile, 75^th^ quartile]

^b^Comparison between the control group, Gly-Fe groups and MHA-Fe groups

^c^Comparison between the control group, 80 mg/kg groups and 115 mg/kg groups

^d^Comparison between the control, 80 mg/kg Gly-Fe, 115 mg/kg Gly-Fe, 80 mg/kg MHA-Fe and 115 mg/kg MHA-Fe groups

^A,B^Different letters in the same row indicate significant differences in the indicators between the treatment groups (*P* < 0.05)

As shown in Table 4, the in situ oscillating sperm ratio was significantly influenced by the source and level of dietary iron. At week 12, the in situ oscillating sperm ratio of boars in the 80 mg/kg or 115 mg/kg MHA-Fe groups was significantly lower than that of the control group. At week 16, there was a trend for the 115 mg/kg MHA-Fe group to have a lower in situ oscillating sperm ratio than the control group (*P* = 0.07). Comparison between groups of iron sources indicated that at weeks 12 and 16 of the study, in situ oscillating sperm ratio was significantly lower in the MHA-Fe group compared to the control group (*P* < 0.05). However, there was no significant difference in the in situ oscillating sperm ratio between the Gly-Fe group and both the MHA-Fe group and the control group. Comparison of iron levels between groups showed that the in situ oscillating sperm ratio of boars in the 115 mg/kg Fe group was significantly lower than that of the control group at weeks 12 and 16 of the study (*P* < 0.05). In addition, at week 16 of the study, the rotational motile sperm ratio was significantly lower in boars in the 115 mg/kg Fe group than in the control and 80 mg/kg Fe groups.

**Table 4:** Effects of different iron sources and levels on sperm motility parameters of boars^a^

Item	Control	Gly-Fe		MHA-Fe		*P*_source_^b^	*P*_level_^c^	*P*_treatment_^d^
		80 mg/kg	115 mg/kg	80 mg/kg	115 mg/kg			
Progressive motile sperm ratio, %								
Week 8	87.55[79.85,92.37]	87.39[83.90,92.00]	81.69[73.18,83.44]	85.50[84.13,88.14]	83.70[80.90,87.20]	0.51	0.07	0.08
Week 12	88.97[76.62,92.74]	89.98[88.58,91.51]	92.08[90.17,93.33]	92.29[89.81,95.23]	92.17[90.04,93.59]	0.06	0.11	0.10
Week 16	89.74[80.60,90.26]	86.52[85.29,90.98]	88.79[86.66,91.07]	88.52[85.19,94.10]	90.68[86.46,93.54]	0.31	0.49	0.55
Fast motile sperm ratio, %								
Week 8	50.10[24.38,59.40]	39.42[29.55,51.88]	40.59[24.00,61.60]	45.79[38.23,54.19]	45.63[33.17,56.63]	0.52	0.99	0.83
Week 12	40.89[29.78,54.46]	41.06[31.09,61.57]	40.67[35.68,64.63]	45.81[36.59,63.13]	48.06[42.39,58.04]	0.49	0.59	0.78
Week 16	30.87[23.13,53.51]	38.77[29.13,50.48]	32.72[28.61,57.87]	39.72[30.45,52.46]	41.84[26.13,47.16]	0.66	0.61	0.87
Slow motile sperm ratio, %								
Week 8	38.26[22.16,57.10]	49.60[33.05,68.50]	44.77[20.35,53.53]	35.76[29.38,43.30]	39.55[21.55,48.37]	0.19	0.93	0.30
Week 12	43.13[34.69,48.49]	43.27[22.01,56.87]	44.30[22.50,51.64]	43.44[23.61,54.10]	40.97[33.68,44.85]	0.99	0.94	0.99
Week 16	50.72[35.84,60.75]	45.41[38.73,56.35]	53.44[24.80,65.60]	53.68[35.82,58.02]	46.69[38.31,55.60]	0.98	0.97	0.98
Rotational motile sperm ratio, %								
Week 8	1.28[0.46,2.43]	1.24[0.25,2.69]	4.29[1.45,5.15]	3.23[1.17,4.26]	1.28[0.73,4.41]	0.43	0.26	0.12
Week 12	1.65[0.94,2.84]	2.35[1.90,4.98]	4.17[2.21,6.33]	2.34[1.54,3.70]	2.13[1.52,4.87]	0.13	0.17	0.24
Week 16	2.98[2.25,4.02]	3.33[2.62,4.68]	2.05[0.90,3.27]	3.70[2.07,4.36]	1.93[1.50,3.80]	0.92	< 0.05	0.16
In situ oscillating sperm ratio, %								
Week 8	2.58[0.83,7.27]	1.12[0.38,5.76]	2.83[1.20,4.16]	2.87[2.42,4.25]	1.55[0.40,2.70]	0.82	0.62	0.22
Week 12	2.44[1.47,6.17]^A^	2.03[0.92,3.96]^AB^	1.18[0.36,2.13]^AB^	0.80[0.29,1.72]^B^	1.20[0.13,1.82]^B^	< 0.01	< 0.01	< 0.05
Week 16	2.93[1.30,5.06]	2.37[1.96,3.34]	2.27[0.27,2.92]	1.65[0.33,4.10]	1.07[0.37,2.76]	< 0.05	< 0.05	0.07

^a^Data was presented as median [25^th^ quartile, 75^th^ quartile]

^b^Comparison between the control group, Gly-Fe groups and MHA-Fe groups

^c^Comparison between the control group, 80 mg/kg groups and 115 mg/kg groups

^d^Comparison between the control, 80 mg/kg Gly-Fe, 115 mg/kg Gly-Fe, 80 mg/kg MHA-Fe and 115 mg/kg MHA-Fe groups

^A,B^Different letters in the same row indicate significant differences in the indicators between the treatment groups (*P* < 0.05)

The effects of different sources and levels of iron supplementation on the morphological parameters of boar sperm were shown in Table 5. At week 12 of the study, the comparison of iron levels between groups showed that the distal protoplasmic droplet ratio of boar sperm in the 115 mg/kg Fe group was lower than that of the control group (*P* < 0.05), but the difference from that of the 80 mg/kg Fe group was not significant. At week 16 of the study, the distal protoplasmic droplet ratio of boar sperm in the 115 mg/kg MHA-Fe group was significantly lower than that of the control group (*P* < 0.05), and the distal protoplasmic droplet ratio of boar sperm in the MHA-Fe group was also significantly lower than that of the control group (*P* < 0.01). In addition, at week 12 of the study, the proximal protoplasmic droplet ratio of boar sperm in the 80 mg/kg MHA-Fe group was significantly lower than that of the control group, and the proximal protoplasmic droplet ratio of boar sperm in the MHA-Fe group was significantly lower than that of the control group (*P* < 0.05). Unexpectedly, at week 8, the folding tail sperm ratio was lower in the 80 mg/kg Fe group than in the 115 mg/kg Fe group, but at week 16, there was no significant difference in the folding tail ratio between boars in the 80 mg/kg Fe group and the 115 mg/kg Fe group, instead, there was a tendency for the folding tail ratio of boars in the 80 mg/kg Fe group to be lower than that of the control group (*P* = 0.06). These results indicated that boars in the 80 mg/kg and 115 mg/kg MHA-Fe groups had lower abnormal sperm rate and in situ oscillating sperm ratio, and better sperm quality compared to the control group. In addition, boars in the 80 mg/kg MHA-Fe group and the 115 mg/kg MHA-Fe group showed different benefits in reducing the rotational motile sperm ratio, folding tail, proximal and distal protoplasmic droplet sperm ratio. However, the effect of Gly-Fe on improving semen quality in boars was not evident.

**Table 5:** Effects of different iron sources and levels on sperm morphological parameters of boars^a^

Item	Control	Gly-Fe		MHA-Fe		*P*_source_^b^	*P*_level_^c^	*P*_treatment_^d^
		80 mg/kg	115 mg/kg	80 mg/kg	115 mg/kg			
Folding tail, %								
Week 8	3.43[0.88,4.84]	2.18[0.98,3.08]	4.46[2.92,5.69]	2.25[1.59,3.88]	3.52[2.56,8.84]	0.72	< 0.05	0.07
Week 12	6.62[1.70,8.60]	2.52[1.16,5.44]	2.42[1.22,6.18]	2.45[0.78,3.64]	2.02[1.71,4.13]	0.16	0.15	0.37
Week 16	6.69[1.23,8.01]	1.52[0.70,3.03]	1.82[0.93,3.07]	1.21[0.68,1.85]	2.82[1.05,5.13]	0.14	0.06	0.17
Distal protoplasmic droplet, %								
Week 8	5.10[3.84,6.78]	3.83[1.60,4.79]	5.41[3.01,9.32]	3.22[2.84,4.88]	2.86[2.40,6.99]	0.22	0.12	0.14
Week 12	6.69[2.45,9.59]	2.86[1.19,6.54]	1.96[1.34,4.27]	2.84[2.53,4.01]	2.44[1.30,4.79]	0.08	< 0.05	0.18
Week 16	4.31[3.43,10.26]^A^	3.48[2.41,6.45]^AB^	3.41[2.73,5.06]^AB^	2.64[2.09,4.03]^AB^	1.95[1.10,3.64]^B^	< 0.01	0.06	< 0.05
Proximal protoplasmic droplet, %								
Week 8	3.38[2.42,4.56]	2.81[1.91,5.42]	3.52[2.75,4.41]	3.00[1.90,3.95]	3.78[2.43,4.34]	0.88	0.31	0.65
Week 12	3.80[2.29,6.22]^A^	2.96[2.01,4.17]^AB^	3.23[1.99,4.11]^AB^	1.55[0.81,2.35]^B^	1.75[1.62,4.57]^AB^	< 0.05	0.17	< 0.05
Week 16	3.25[1.99,5.16]	2.89[1.23,3.46]	3.66[2.17,5.52]	2.33[1.98,3.36]	2.15[1.34,3.05]	0.22	0.76	0.29

^a^Data was presented as median [25^th^ quartile, 75^th^ quartile]

^b^Comparison between the control group, Gly-Fe groups and MHA-Fe groups

^c^Comparison between the control group, 80 mg/kg groups and 115 mg/kg groups

^d^Comparison between the control, 80 mg/kg Gly-Fe, 115 mg/kg Gly-Fe, 80 mg/kg MHA-Fe and 115 mg/kg MHA-Fe groups

^A,B^Different letters in the same row indicate significant differences in the indicators between the treatment groups (*P* < 0.05)

### Pro-inflammatory cytokine levels affect oxidative stress and body iron status, modulating boar semen quality

**Fig. 4:**
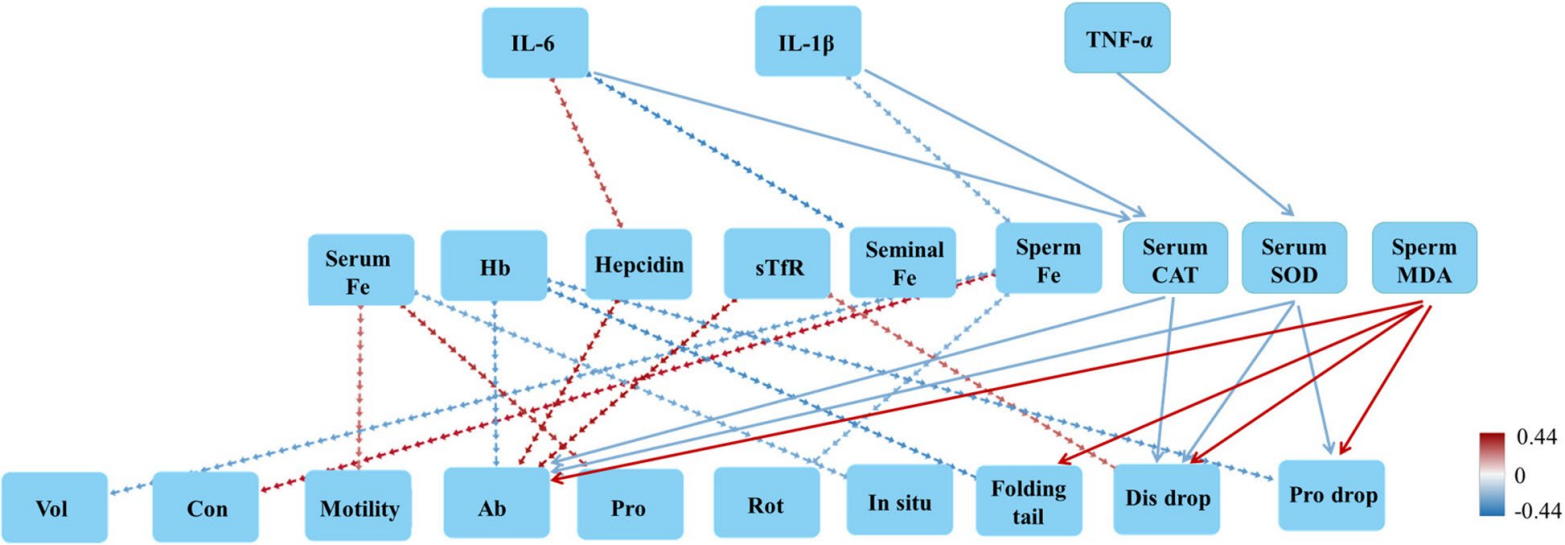
Correlation between serum inflammatory factor levels, iron status indicators, oxidative stress levels, and semen quality parameters in boars. Hb: hemoglobin; sTfR: serum transferrin receptor; Vol: ejaculate volume; Con: sperm concentration; Ab: abnormal sperm rate; Pro: progressive motile sperm ratio; Rot: rotational motile sperm ratio; In situ: in situ oscillating sperm ratio; Dis drop: distal protoplasmic droplet; Pro drop: proximal protoplasmic droplet. The dotted line represents the relationship between iron status indicators and boar inflammatory factor levels, as well as semen quality, while the solid line illustrates the relationship between oxidative stress indicators, inflammatory factor levels, and semen quality. A significant negative correlation was denoted by the blue line, and a significant positive correlation was indicated by the red line (*P* < 0.05)

As shown in Fig. 4, correlation between serum inflammatory factor levels, iron status indicators, oxidative stress levels, and semen quality parameters in boars revealed that a positive correlation between serum IL-6 levels and hepcidin concentration (*P* < 0.05), with an increase in hepcidin concentration associated with elevated abnormal sperm rate (*P* < 0.05). In addition, serum IL-6 level was significantly negatively correlated with seminal plasma Fe concentration (*P* < 0.05). Serum IL-1β level was significantly negatively correlated with sperm Fe concentration (*P* < 0.05), while there was a correlation between decreased sperm Fe concentration and increased rotational motile sperm ratio (*P* < 0.05). The results of correlation between indicators of iron status and semen quality parameters showed that serum Fe level was positively correlated with sperm motility and progressive motile sperm ratio, and negatively correlated with in situ oscillating sperm ratio (*P* < 0.05). Hemoglobin concentration was significantly negatively correlated with abnormal sperm rate, folding tail and proximal protoplasmic droplet content (*P* < 0.05). However, serum transferrin receptor level was significantly positively correlated with abnormal sperm rate and distal protoplasmic droplet content (*P* < 0.05). In addition, IL-6 and IL-1β were negatively correlated with serum CAT content, and TNF-α was negatively correlated with serum SOD content (*P* < 0.05). Further, serum CAT and SOD contents were negatively correlated with abnormal sperm rate, proportion of distal protoplasmic droplet and/or proximal protoplasmic droplet (*P* < 0.05). Sperm MDA content was positively correlated with abnormal sperm rate, folding tail, proportion of distal and proximal protoplasmic droplets (*P* < 0.05), although the correlation with inflammatory cytokines was not significant.

## Discussions

Iron is an essential trace element for maintaining normal spermatogenesis and excellent semen quality [18]. Therefore, different sources and levels of dietary iron may modulate boar semen quality by influencing boar body iron levels and iron status. In this study, we found that dietary supplementation with either 80 mg/kg or 115 mg/kg of MHA-Fe did not lead to reduced serum Fe and hemoglobin levels. On the contrary, it resulted in decreased levels of serum IL-6 and hepcidin, further ameliorated oxidative stress, increased body iron utilization, and improved semen quality in adult boars.

### No reduction in serum iron and hemoglobin levels in boars fed 80 mg/kg or 115 mg/kg Gly-Fe and MHA-Fe

Iron in the serum consists mainly of iron released by macrophages phagocytosing senescent or damaged erythrocytes and dietary iron absorbed in the duodenum, which is subsequently transported with the circulation to various tissues and organs for utilization [19]. Studies have shown that the order of priority of iron delivery to tissues and organs is erythrocytes > brain > heart > liver > skeletal muscle [20], suggesting that use for hemoglobin synthesis in erythrocytes is the most principal function of iron and is extremely important for the vital activities of the organism. In the present study, we observed that dietary iron treatments from different sources and levels did not affect boar serum iron and hemoglobin concentrations, which means that reducing the total iron levels to 80 mg/kg or 115 mg/kg when feeding organic iron to boar diets does not lead to iron deficiency and affect hemoglobin synthesis in boar, and that it is feasible and can be practically applied in production.

Hepcidin, a hormone secreted by hepatocytes to regulate iron homeostasis [21], is essentially an antimicrobial peptide elevated in high serum iron or inflammatory states [22], which is able to reduce serum iron levels by degrading ferroportin on the membranes of intestinal epithelial cells and macrophages, thereby inhibiting iron absorption in the intestine and recycling iron from the macrophages into the blood [23]. Compared with the control group fed 150 mg/kg of mixed iron sources, boars in the other four treatment groups had reduced serum hepcidin levels, indicating increased intestinal iron absorption and macrophage iron influx, and stabilization of body iron levels after dietary iron levels were reduced. In addition, hepcidin may have been further reduced and body iron levels increased when the inflammatory state of boars in the 80 mg/kg or 115 mg/kg MHA-Fe group was alleviated. In short, when organic iron is the dietary source, reducing iron levels does not cause boar iron deficiency, and selecting an appropriate organic source may further increase iron concentrations by reducing inflammation and hepcidin levels.

### Reduced inflammatory response alleviates oxidative stress and improves body iron utilization in boars fed 80 mg/kg or 115 mg/kg MHA-Fe

Compared to the control group fed 150 mg/kg of mixed iron sources, the inflammatory response and oxidative stress state of boars in the 80 mg/kg or 115 mg/kg MHA-Fe groups were alleviated. The reason may be mainly due to the differences in the amount of free Fe^2+^ released from different iron sources and its induced Fenton reaction, which caused varying amounts of ROS [24]. The absorption of FeSO_4_ in the duodenum is only 5%–15%, the unabsorbed FeSO_4_ in the gastrointestinal tract may generate a large amount of ROS due to the susceptibility of Fe^2+^ to oxidation in the presence of oxygen, leading to oxidative stress and causing intestinal inflammation [25]. Furthermore, the inflammatory response as an immune process further promotes the production of ROS and increases the level of oxidative stress [26]. Hence, boars fed 80 mg/kg or 115 mg/kg MHA-Fe may experience a reduction in the amount of free Fe^2+^, leading to decreased levels of ROS and IL-6, thereby alleviating oxidative stress. This effect could be attributed to the high stability and absorption of MHA-Fe in the gastrointestinal tract. In particular, the pro-inflammatory cytokine IL-6 may alter the body iron status by regulating hepcidin levels. Ferritin, an indicator of the body iron stores [27], is an acute temporal reactant that is also elevated in inflammatory states [15, 28]. Serum transferrin receptor is the cleaved monomer of tissue transferrin receptor, and its concentration is proportional to the total amount of transferrin receptor in tissues. It mainly reflects the amount of iron available to the organism as well as the amount of iron required. Therefore, its content in serum increases when the available iron is reduced or the amount of iron required is increased [29]. Our research findings indicate that the levels of ferritin and transferrin receptor in boars fed 80 mg/kg or 115 mg/kg MHA-Fe decreased, along with decreased serum IL-6 and hepcidin levels. This reflects the possibility that dietary supplementation with an appropriate iron source, even at low levels, may reduce body iron stores and increase the amount of available iron.

Studies on the effects of different dietary Fe levels on spermatogenesis and sperm quality in male animals have been reported [18, 30]. However, in these studies, researchers have rarely examined the levels of Fe in serum, seminal plasma, and sperm. In our study, we observed a significant increase in seminal plasma Fe content in boars fed with 115 mg/kg MHA-Fe compared to the control group, despite no significant difference in serum Fe content and sperm Fe content. This suggests an increase in the Fe content in the secretions of the prostate, seminal vesicles, and bulbourethral glands. However, accurately measuring the Fe content reaching the testes and accessory glands of boars using existing blood and semen samples poses challenges. Accurately assessing the effects of dietary Fe treatment on body iron levels, iron status, Fe content in tissues and organs, and Fe content in germ cells is crucial for understanding the regulation of spermatogenesis and semen quality by Fe. This also provides direction for our future research endeavors. In summary, these data suggest that feeding boars with 80 mg/kg or 115 mg/kg MHA-Fe resulted in reduced serum inflammatory response and hepcidin levels. This, in turn, alleviated oxidative stress and improved body iron utilization, potentially leading to increased iron transfer to the male reproductive organs.

### Dietary supplementation with 80 mg/kg or 115 mg/kg MHA-Fe improved boar semen quality

Dietary supplementation with iron, copper and other complex organic micronutrients has been reported to increase fertility [31], improve semen quality and slow testicular degeneration in bulls compared to inorganic micronutrients [32]. In the present study, it was found that the addition of 80 mg/kg or 115 mg/kg MHA-Fe to the diet significantly improved sperm motility and reduced abnormal sperm rate in boars as compared to the control group fed 150 mg/kg of mixed iron sources. However, the addition of 80 mg/kg or 115 mg/kg Gly-Fe to the diets did not significantly improve boar semen quality, which may be related to the form of Gly-Fe absorbed in the intestinal epithelium. Gly-Fe enters the gastrointestinal tract bound to amino acids, which reduces the chances of Fe^2+^ being bound by antagonists and forming insoluble compounds in the gastrointestinal tract, thus increasing the quantity of Fe^2+^ reaching the intestinal epithelial cells [33, 34]. However, similar to FeSO_4_, Gly-Fe is primarily absorbed by intestinal epithelial cells in the form of Fe^2+^, resulting in a high concentration of free Fe^2+^ still present at the intestinal epithelial cells [35, 36]. This elevated level of Fe^2+^ may lead to an increase in the production of ROS [24]. The intestinal absorption form of MHA-Fe is mainly an amino acid complex, which may have reduced free Fe^2+^. Accordingly, we found that the levels of serum IL-6 and serum, seminal plasma and sperm MDA were higher in boars in the Gly-Fe and mixed iron groups than in the MHA-Fe group. In addition, serum IL-6 level was positively correlated with hepcidin concentration, which correlated with the increased abnormal sperm rate. The concentration of MDA, a product of oxidative stress, was negatively correlated with sperm viability, sperm density and sperm morphology [37]. Adding 80 mg/kg or 115 mg/kg of MHA-Fe to the diet effectively improved the semen quality of boars. This suggests that, when the iron addition meets the basic requirements, the appropriate source of iron is a key factor affecting the semen quality of boars. Inappropriate mixed iron supplementation patterns, even as high as 150 mg/kg, may adversely affect boar semen quality.

## Conclusions

Compared to the conventional nutritional regimen of mixing iron sources at 150 mg/kg, adding 80 mg/kg or 115 mg/kg of MHA-Fe to the diet did not decrease serum iron and hemoglobin levels in boars; instead, it significantly improved semen quality. This improvement may be associated with reduced inflammation and oxidative stress levels, as well as increased iron utilization. However, a key question remains unanswered in current research: whether dietary iron from different sources or levels alters the distribution of iron in various tissues and organs, including the testes, by affecting body iron status, thereby influencing the level and function of germ cell iron and ultimately regulating boar semen quality. Clarifying the pathway of iron from diet to the bloodstream, to germ cells in the testes, and to accessory glands is crucial for understanding the regulation of spermatogenesis and semen quality by iron elements. It also provides theoretical support for developing nutritional regulation techniques to enhance boar semen quality.

## Data Availability

The datasets during and/or analyzed during the current study available from the corresponding author on reasonable request.

## Abbreviations

CASA: Computer-assisted sperm testing system
CAT: Catalase
Fe: Iron
FeSO_4_: Ferrous sulfate
Gly-Fe: Glycine chelated iron
IL-10: Interleukin-10
IL-1β: Interleukin-1β
IL-6: Interleukin-6
MDA: Malondialdehyde
MHA-Fe: Methionine hydroxyl analogue chelated iron
ROS: Reactive oxygen species
SOD: Superoxide dismutase
T-AOC: Total antioxidant capacity
TNF-α: Tumor necrosis factor-α
